# Novel anti-obesity effects of beer hops compound xanthohumol: role of AMPK signaling pathway

**DOI:** 10.1186/s12986-018-0277-8

**Published:** 2018-06-15

**Authors:** Janaiya S. Samuels, Rangaiah Shashidharamurthy, Srujana Rayalam

**Affiliations:** Department of Pharmaceutical Sciences, School of Pharmacy, Philadelphia College of Osteopathic Medicine, 625 Old Peachtree Rd NW, Suwannee, GA 30024 USA

**Keywords:** Xanthohumol, 3T3-L1 cells, Beiging, Obesity, AMPK signaling, UCP1, Lipolysis, Thermogenesis, Oxygen consumption rate, Human primary subcutaneous adipocytes, ACC

## Abstract

**Background:**

Obesity alters adipose tissue metabolic and endocrine functioning, leading to an increased adiposity and release of pro-inflammatory cytokines. Various phytochemicals have been reported to contribute to the beiging of white adipose tissue in order to ameliorate obesity by increasing thermogenesis. Here, we show that the prenylated chalcone, xanthohumol (XN), induces beiging of white adipocytes, stimulates lipolysis, and inhibits adipogenesis of murine 3T3-L1 adipocytes and primary human subcutaneous preadipocytes and these effects are partly mediated by the activation of the AMP-activated protein kinase (AMPK) signaling pathway.

**Methods:**

3T3-L1 adipocytes and primary human subcutaneous preadipocytes were differentiated using a standard protocol and were treated with various concentrations of XN, dorsomorphin, an AMPK inhibitor, or AICAR, an AMPK activator, to investigate the effects on adipogenesis, beiging and lipolysis.

**Results:**

XN induced beiging of white adipocytes as witnessed by the increased expression of beige markers CIDE-A and TBX-1. XN increased mitochondrial biogenesis, as evidenced by increased mitochondrial content, enhanced expression of PGC-1α, and the thermogenic protein UCP1. Following 24 h of treatment, XN also increased oxygen consumption rate. XN stimulated lipolysis of mature 3T3-L1 and primary human subcutaneous adipocytes and inhibited adipogenesis of maturing adipocytes. XN activated AMPK and in turn, XN-induced upregulation of UCP1, p-ACC, HSL, and ATGL was downregulated in the presence of dorsomorphin. Likewise, an XN-induced decrease in adipogenesis was reversed in the presence of dorsomorphin.

**Conclusions:**

Taken together, XN demonstrates anti-obesity effects by not only inducing beiging but also decreasing adipogenesis and inducing lipolysis. The anti-obesity effects of XN are partly mediated by AMPK signaling pathway suggesting that XN may have potential therapeutic implications for obesity.

**Electronic supplementary material:**

The online version of this article (10.1186/s12986-018-0277-8) contains supplementary material, which is available to authorized users.

## Background

Obesity develops when energy intake, in the form of food, exceeds energy expenditure [1] and is primarily characterized by excessive adiposity [2]. There are two functionally and morphologically distinct types of adipose tissue: white adipose tissue (WAT) and brown adipose tissue (BAT), both of which are mediators of energy homeostasis. WAT functions to store energy in the form of lipids in white adipocytes and secretes adipokines amongst other factors including leptin, adiponectin, tumor necrosis factor α (TNF-α), to regulate energy metabolism and immunometabolism [3, 4]. BAT functions as an energy dissipating, thermogenic adipose tissue because of its enhanced mitochondrial content and uncoupling protein 1 (UCP1) [5]. In response to specific stimuli, such as beta-adrenergic stimulation or cold exposure, WAT can acquire brown-like characteristics. This process is referred to as “beiging”. Differentiation of WAT to beige adipose tissue (BeAT) has been demonstrated in vivo [6] and in vitro [7] to improve the metabolic profile and increase thermogenesis.

Many phytochemicals such as guggulsterone, curcumin, and resveratrol inhibit adipogenesis, stimulate lipolysis in adipocytes, and have been recently proposed to act as beiging agents [7–9]. The mechanisms driving these responses are not as well defined. Minimal attention has been directed towards exploring the beiging potential of phytochemicals or the molecular mechanisms behind it. A recent study demonstrated that 3T3-L1 adipocytes are a useful model to study lipid droplet formation and adipogenesis [10]. Thermogenesis has been shown to decrease adiposity and ameliorate diabetes in both mice and adult humans [11–13].

Following this model, the current study sought to investigate if the anti-obesity phytochemical, xanthohumol (XN), a prenylated flavonoid extracted from the hops plant *Humulus lupus,* can drive mitochondrial biogenesis and beiging in 3T3-L1 and human subcutaneous adipocytes. We also sought to determine if XN’s anti-obesity effects are mediated through the adenosine-monophosphate protein kinase (AMPK) signaling pathway. Our in vitro and in vivo data provides novel evidence of XN’s role in the modulation of improving the metabolic profile.

AMPK is a master metabolic switch and is believed to be expressed in a number of tissues including BAT. The thermogenic activities of BAT are activated by AMPK [14]. BAT activation is stimulated in response to cold conditions resulting in the phosphorylation of AMPK to begin to increase thermogenesis. AMPK is an enzyme complex composed of three subunits, the catalytic α subunit and the two regulatory β and γ subunits. The α1 isoform is the predominant isoform expressed in adipose tissue [15]. AMPK is phosphorylated at Thr172 of the α subunit and as a result, becomes biologically active [16].

In our metabolic organs that function to maintain energy homeostasis, like adipose tissue, phosphorylated-AMPK (p-AMPK) inhibits anabolic processes and upregulates catabolic processes. p-AMPK works to enhance fatty acid oxidation by the phosphorylation of acetyl-CoA carboxylase (ACC), thereby increasing the incidence of fatty acid degradation [17].

AMPK’s role in adipose tissue metabolism has been well characterized [18] and studies have demonstrated that flavonoids like curcumin and resveratrol-induced beiging of adipocytes is dependent upon AMPK activation [4, 7]. Xanthohumol has also been demonstrated to activate AMPK [19] but its correlation between beiging and AMPK activation remains unknown. Therefore, in this study, we aimed to investigate the potential relationship between XN-mediated beiging and AMPK signaling.

XN has been shown to possess anti-carcinogenic, anti-oxidant, and anti-diabetic properties [20–22]. It has been suggested that the chemical structure of XN is responsible for its wide range of biological activities. The XN molecule is comprised of two aromatic rings substituted with hydroxyl and methoxyl groups, and a prenyl unit. Because of its prenyl and – OCH3 group, XN is highly lipophilic and has a strong affinity for biological membranes [23]. Hirata et al., reported that XN inhibits the cholesteryl ester transfer protein (CETP), resulting in an increase in high density lipoprotein levels. This inhibitory effect can be attributed to XN’s prenyl group and chalcone structure [24]. Costa et al., demonstrated that XN consumption in high-fat diet fed mice prevented weight gain, decreased blood glucose levels, triglyceride, and cholesterol levels, and improved insulin sensitivity [25]. XN also activated AMPK signaling pathway, suppressing lipogenesis [25]. Oral administration of XN improved inflammatory markers and the metabolic profile in diet-induced obese C57BL/6 J mice [26]. However, XN’s beiging effects on adipocytes and the underlying mechanisms remain to be understood.

## Methods

### Cell culture of 3T3-L1 adipocytes

Dulbecco’s Modification of Eagle’s Medium (DMEM; Corning, Manassas, VA, USA) supplemented with 10% calf serum (CS; Invitrogen, Grand Island, NY, USA) and 1% penicillin streptomycin (PS; Sigma-Aldrich, St. Louis, MO, USA) was used to culture the 3T3-L1 mouse embryo preadipocyte cell line (Zenbio, Research Triangle Park, NC, USA) at 37 °C in a 5% CO2 incubator. Two day post confluent preadipocytes were maintained in differentiation induction medium I (DM I) comprised of 1 mg/ml of insulin (Ins; Sigma-Aldrich, St. Louis, MO, USA), 5 μM of dexamethasone (Dexa; Sigma-Aldrich, St. Louis, MO, USA), 0.5 mM of isobuytlmethylxanthine (IBMX; Sigma-Aldrich, St. Louis, MO, USA), and 1 μM of rosiglitazone (Rosi; Sigma-Aldrich, St. Louis, MO, USA) for 3 days in DMEM plus 10% fetal bovine serum (FBS; Invitrogen, Grand Island, NY, USA) and 1% PS. Following DM I, cells were then maintained in differentiation medium II (DM II) containing 10% FBS, 1% PS, 1 mg/ml of Ins, and 1 μM of Dexa. Unless otherwise stated, cells were maintained in DM II for 4–6 days before treatment and analysis and until cells matured with a minimum of 90% lipid droplet accumulation. DM II maintenance medium was changed every other day. XN 6.25 μM and XN 25 μM were the two optimal doses used in this study based on cell viability assays. To test the effects of 5-Amionimidazole-4-carboxamide (AICAR; Santa Cruz Biotechnology, Dallas, TX, USA) and dorsomorphin (Dorso; Abcam, Cambridge, MA, USA) on AMPK activity, mature 3T3-L1 adipocytes were treated with AICAR 2 mM or dorsomorphin 10 μM for one hour.

### Cell culture of primary human subcutaneous adipocytes

Primary human subcutaneous preadipocytes were purchased from Lonza (Alpharetta, GA, USA). Preadipocytes were cultured in preadipocyte growth medium (PGM) supplemented with 10% FBS, 2 mM L-glutamine, 30 μg/mL of gentamycin sulfate, and 15 ng/mL of amphotericin B (Lonza, Alpharetta, GA, USA). Differentiation of human subcutaneous adipocytes was initiated using complete PGM supplemented with insulin, dexamethasone, indomethacin, rosiglitazone, and IBMX (replenished every other day; Lonza, Alpharetta, GA, USA). Adipocytes were matured for 10 days before stimulation with XN.

### Cytotoxicity

Confluent mature 3T3-L1 and primary human subcutaneous adipocytes were treated with 0.1% DMSO vehicle control or XN for 24 to 96 h. Cell viability was measured using the Prestoblue™ Cell Viability Reagent according to the manufacturer’s protocol. The absorbance of metabolically active cells were quantified 1 h after incubation in the reagent using the Biotek Synergy HT microplate reader at 570 nm.

### Immunoblot analysis

Cell lysates were prepared using ice cold RIPA Lysis and Extraction buffer, complete with protease and phosphatase inhibitors (ThermoFisher Scientific, Grand Island, NY, USA). Following protein estimation, as determined by the Pierce BCA Protein Assay Kit (ThermoFisher Scientific, Grand Island, NY, USA), cell extract was diluted in 4X sample buffer and heated for 5 min at 95 °C before 4–20% sodium dodecyl sulfate-polyacrylamide gel electrophoresis. After electrophoresis, samples were transferred to a polyvinylidene difluoride (PVDF) membrane using a Trans-blot Turbo system (Bio-Rad, Hercules, California, USA) and then blocked for 1 h with Tris buffered saline plus 0.1% Tween 20 (TBS-T) containing 5% bovine serum albumin (BSA). Following blocking, the membrane was incubated for 1 h with 1:1000 dilutions in primary antibodies, anti-UCP1, anti-CIDE-A, anti-TBX1, anti-phospho-AMPK (Thr172), anti-phospho-ACC (Ser79), anti-AMPK (α1/2), (all from Abcam, Cambridge, MA, USA), anti-PGC-1α, anti-ZIC1, (Novus Biologicals, Littleton, CO, USA), and anti-β-actin (Santa Cruz Biotechnology, Cambridge, MA, USA). After three washes with TBS-T buffer, the membrane was incubated for 45 min at room temperature with secondary antibodies conjugated to IRDye 800 and developed using the Odessey CLX imaging system (LiCor Biosciences, Lincoln, NE, USA). Relative protein levels were quantified using Image Studio Ver. 5.2 (LiCor Biosciences, Lincoln, NE, USA).

### Mitochondrial content

Mature 3T3-L1 adipocytes were treated with 0.1% DMSO, XN 6.25 μM, XN 25 μM, and Iso 10 μM for 24 h and incubated with Mitotracker® Green FM (Invitrogen, Grand Island, NY, USA) as per the manufacturer’s instructions. Mitochondrial activity was measured using the Biotek Synergy HT (Winooski, VT, USA) microplate reader at 516 nm.

### Oil red O staining

DMSO and XN treated cells were induced to differentiate for 4–8 days, followed by washing with phosphate buffered saline (PBS), fixation with 10% formalin for 1 h at room temperature, and washing again three times with deionized water. A 6:4 mixture of Oil Red O solution (0.6% Oil Red O dye in isopropanol) and water was added to the cells for 20 min followed by a wash four times with deionized water. Finally, hematoxylin was layered over the cells and incubated for one minute, then rinsed. Lipid droplets were imaged under phase contrast using the EVOS FL Auto Imaging System (ThermoFisher Scientific, Grand Island, NY, USA) microscope. The Oil Red O staining is a biological agent used only for the assessment of lipid content. Therefore, it does not quantitatively measure total cell number.

### AdipoRed™ adipogenesis assay

Both 3T3-L1 and primary human subcutaneous preadipocytes were treated with 0.1% DMSO, XN 6–25 μM, dorsomorphin 10 μM or AICAR 2 mM during the adipogenesis period for 8–10 days and the lipid accumulation was quantified using the AdipoRed™ assay (Lonza, Alpharetta, GA, USA) per the manufacturer’s protocol. AdipoRed is a solution of the hydrophilic stain Nile Red. Mature adipocytes treated with 0.1% DMSO, XN 25 μM, dorsomorphin 10 μM, or AICAR 2 mM for 72 h were also assayed on day 10 for lipid quantification. Lipid quantification was obtained using the Biotek Synergy HT (Winooski, VT, USA) microplate reader at a fluorescence of 485/590 nm. Lipid content to the number of cells present in each well was normalized for the AdipoRed™ adipogenesis assay.

### Oxygen consumption rate

3T3-L1 premature adipocytes were seeded into a 96-well plate at a density of 10,000 cells/well. Cells were grown to confluence and differentiated into mature adipocytes as per the described protocol above. Mature adipocytes were treated with XN 6.25 or 25 μM or isoproterenol 10 μM for 24 h prior to the start of the Oxygen Consumption Rate Assay Kit, MitoXpress® Xtra HS Method (Cayman Chemical, Ann Arbor, MI, USA). Antimycin A 1 μM was used as a negative control to determine oxygen consumption rate (OCR). Time-resolved fluorescence was obtained over an hour and twenty minutes.

### Statistical analysis

All data were expressed as the mean ± SEM. All assays were performed in triplicate for a minimum of three independent experiments. Comparisons were made by using One-way ANOVA on GraphPad Prism software, followed by Tukey’s post hoc tests. Statistical significance was reported as * *P* < 0.05 or ** *P* < 0.01. Means denoted with different letters are representations of comparisons between each treatment groups at a *P* < 0.05 significance level.

## Results

### XN does not produce cytotoxic effects in 3T3-L1 adipocytes

**Fig. 1:**
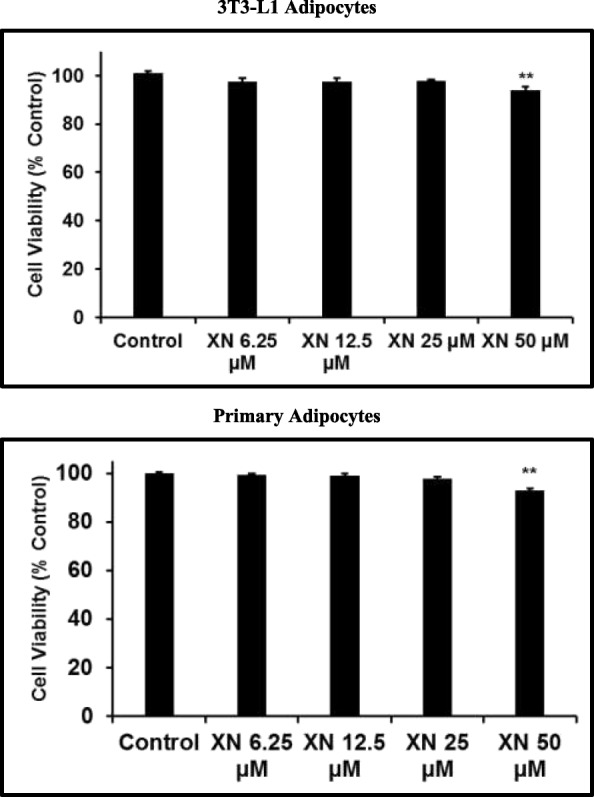
XN did not induce cytotoxicity in 3T3-L1 and primary human adipocytes. XN 6.25–25 μM treatment for 24 to 96 h in mature, 3 T3-L1 adipocytes did not induce cytotoxicity. XN stimulation of primary human subcutaneous adipocytes did not alter cell viability at 6.25–25 μM. All data are presented as mean ± SEM. Statistical significance between control and treatment groups is depicted as ** *P* < 0.01

Mature 3T3-L1 and primary human subcutaneous adipocytes were treated with 0.1% DMSO, XN 6–50 μM for 24 to 96 h. Following incubation for 24 h, XN 6.25–25 μM was not significantly cytotoxic to adipocytes in both cell types (Fig. 1 and Additional file 1). Kirkwood et al., demonstrated that low concentrations of XN acutely increases uncoupled respiration while higher doses inhibited respiration [27]. Additionally, the plasma levels of XN in HFD rats fed low (1.86 mg/kg BW) and medium (5.69 mg/kg BW) doses of XN achieved 50 ± 14 and 107 ± 46 nM respectively (*p* > 0.05), while high dose of XN (16.9 mg/kg BW) achieved significantly higher levels of plasma XN (389 ± 153 nM) [28]. While it is difficult to translate in vivo doses to in vitro doses, based on previous in vitro studies published and our current cell viability data, we have selected low (6.25 μM) and high (25 μM) concentrations of XN for the subsequent experiments.

### XN induces beiging of mature 3T3-L1 and increases mitochondrial biogenesis

**Fig. 2:**
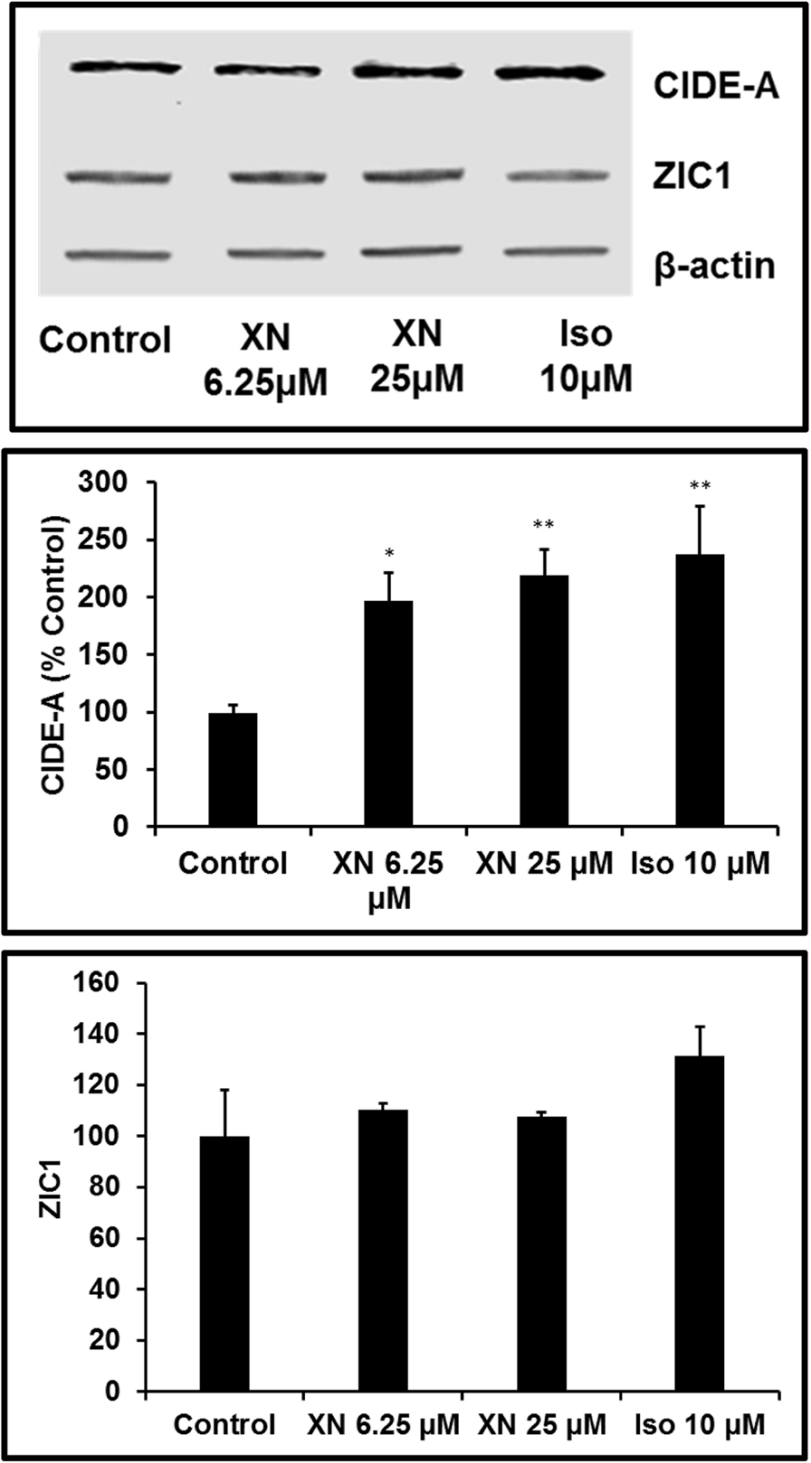
XN treatment induces expression of brown/beige fat markers. XN stimulation of 3T3-L1 and upregulates beige markers CIDE-A and ZIC1. All data are presented as mean ± SEM. Statistical significance between control and treatment groups is depicted as * *P* < 0.05, and ** *P* < 0.01

**Fig. 3:**
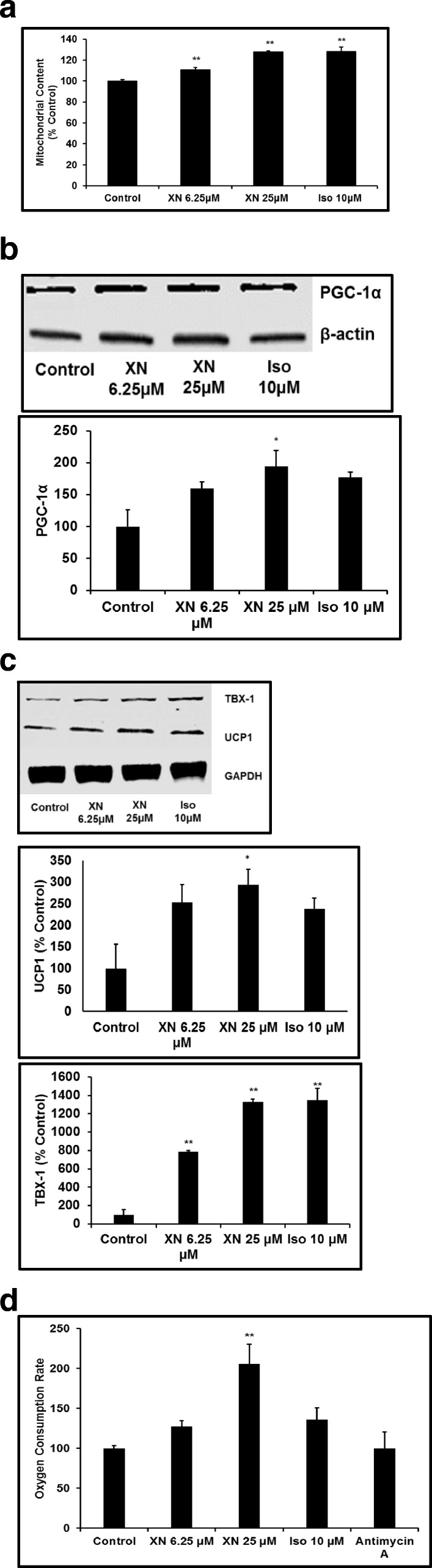
XN increases mitochondrial content, oxygen consumption rate, and upregulates thermogenic marker expression in 3T3-L1 adipocytes. **a**. Various doses of xanthohumol (6.25 and 25 μM) were incubated with mature, 3T3-L1 adipocytes, followed by quantification of mitochondrial content using MitoTracker® Green FM assay. All data are presented as the mean ± SEM. Means denoted with different letters are statistically different, *P* < 0.05. **b**. Protein expression levels of PGC-1α, a regulator of mitochondrial metabolism in 3T3-L1 adipocytes after 24 h of treatment with XN or isoproterenol. **c**. UCP1 and TBX1 protein expression levels post-24 h XN or Iso stimulation. **d**. OCR as determined by the Oxygen Consumption Rate Assay Kit following 24 h of treatment with XN or isoproterenol. All data are presented as mean ± SEM. Statistical significance between control and treatment groups is depicted as *P < 0.05, **P < 0.01

To investigate the beiging effect of XN, 3T3-L1 adipocytes were stimulated with XN 6.25–25 μM and isoproterenol 10 μM, as a positive control. XN significantly increased the expression of the beige adipocyte specific marker CIDE-A in a dose-dependent manner (Fig. 2). ZIC1, a classic brown adipocyte marker, showed no significant upregulation when 3T3-L1 adipocytes were stimulated with XN 6.25–25 μM (Fig. 2), suggesting XN-induced beiging. To determine if XN increases mitochondrial biogenesis, a characteristic of the beiging of white adipocytes, MitoTracker® Green was conducted. As shown in Fig. 3a, mitochondrial content was markedly elevated in XN-treated groups and this was further confirmed by Western blot analysis of PGC-1α, a central driver of mitochondrial biogenesis in adipocytes (Fig. 3b). Furthermore, increased expression of the mitochondrial uncoupling protein 1 (UCP1, Fig. 3c), a thermogenic marker, an increase in OCR (Fig. 3d), and a strikingly significant upregulation of the beige adipocyte marker, TBX-1 (Fig. 3c), strongly support the role of XN in driving the induction of the beiging of white adipocytes.

### XN regulates lipid metabolism in 3T3-L1 adipocytes

**Fig. 4:**
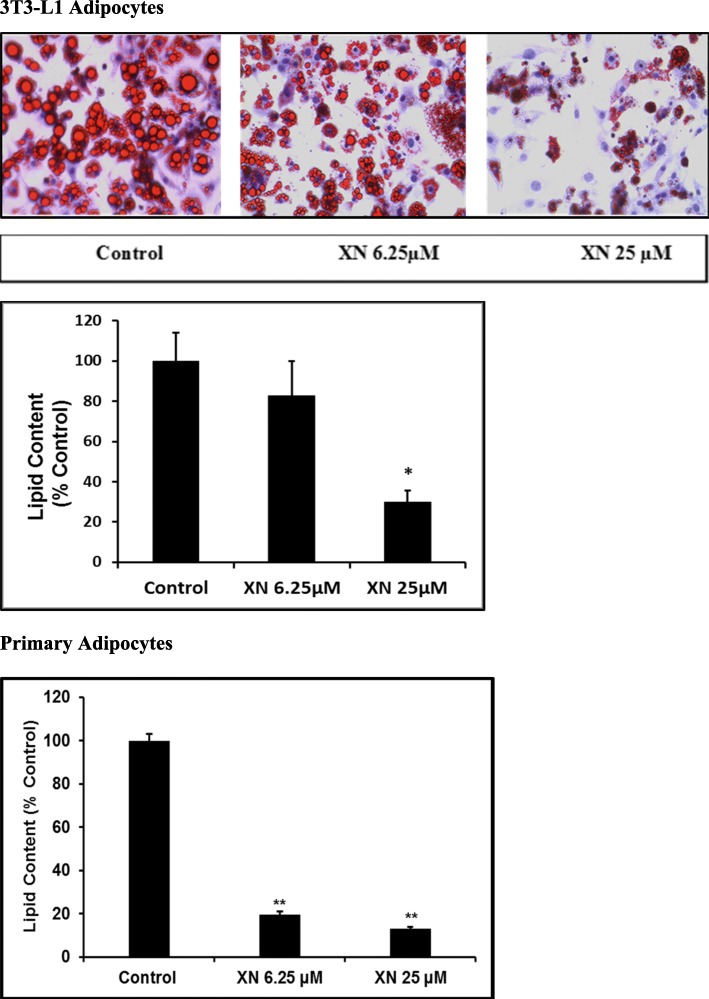
XN inhibits preadipocyte differentiation and adipogenesis. Oil Red O staining was performed to assess the maturation of 3T3-L1 adipocytes. AdipoRed™ assay was used to quantitate XN-induced suppression of lipid accumulation in 3T3-L1 adipocytes and primary human subcutaneous adipocytes. All data are presented as mean ± SEM. Statistical significance between control and treatment groups is shown as * *P* < 0.05, ** *P* < 0.01

To establish XN’s multi-faceted anti-obesity effects on 3T3-L1 and primary human subcutaneous adipocytes, we examined whether XN inhibits preadipocyte differentiation. Preadipocytes were treated with XN 6.25–25 μM and 0.1% DMSO vehicle control on day 0 until day 7 of differentiation. As visualized by Oil Red O staining (Fig. 4), XN decreased the number and size of lipid droplets in both 3T3-L1 and primary human subcutaneous adipocytes, suggesting that XN inhibits adipogenesis and suppresses lipid accumulation. Lipid content was normalized to cell number as assessed by cell viability assay.

### XN stimulates the phosphorylation of AMPK in mature 3T3-L1 adipocytes

**Fig. 5:**
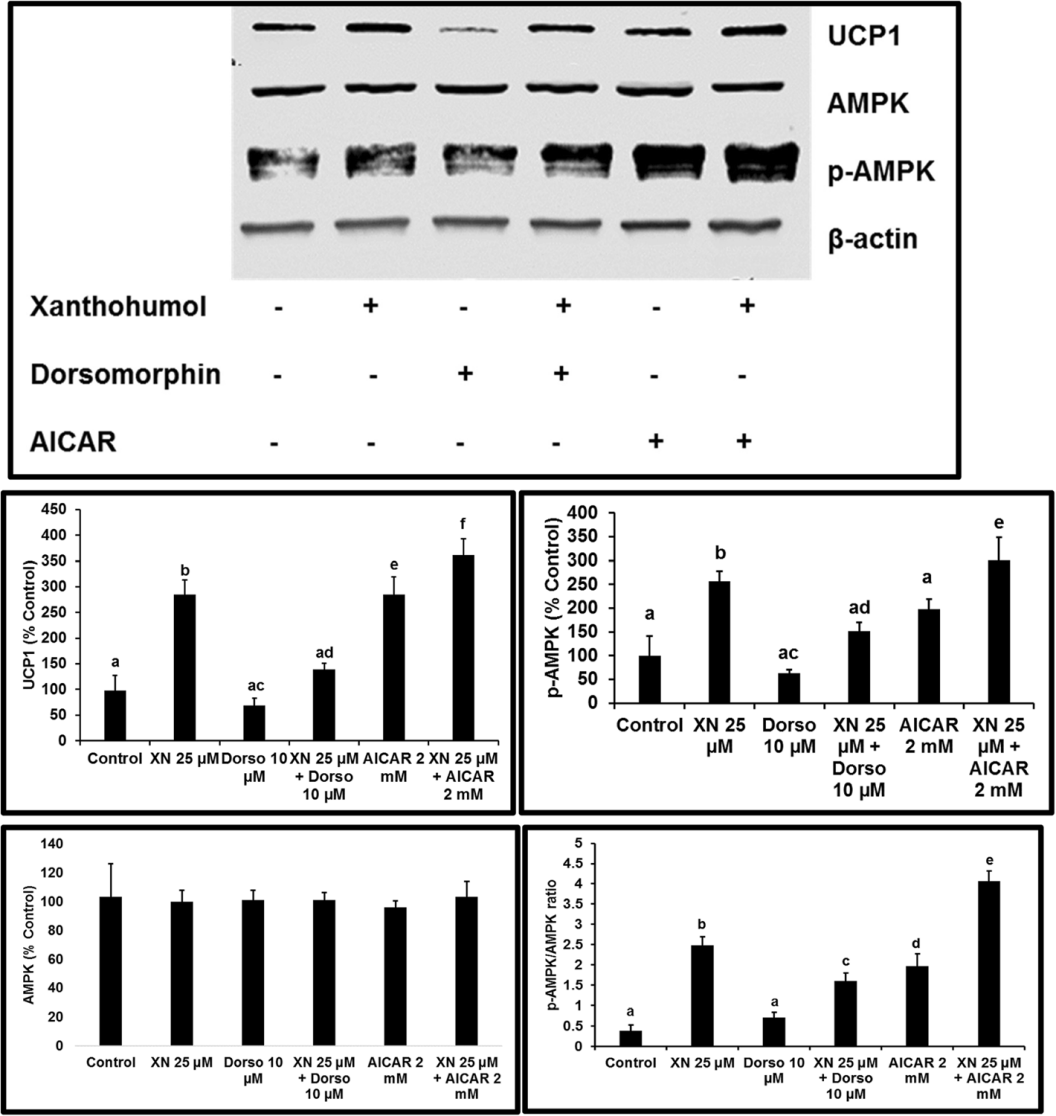
Effect of XN, dorsomorphin, and AICAR on AMPK activation and expression of UCP1. Mature adipocytes were treated for one hour and p-AMPK, total AMPK, and UCP1 protein expression levels were measured. All data are presented as the mean ± SEM. Means denoted with different letters are statistically different, *P* < 0.05

*A*MPK is an important regulator of metabolic homeostasis within the cell [29, 30]. A consequence of the activation of AMPK signaling is lipid metabolism modulation, mitochondrial biogenesis, and a decrease in blood glucose levels [30]. Therefore, we sought to investigate the effect of XN on AMPK phosphorylation. XN treatment of mature 3T3-L1 adipocytes significantly activated AMPK as evidenced by the enhanced expression levels of p-AMPK, similar to that of the AMPK agonist AICAR (Fig. 5).

### Inhibition of AMPK decreases the thermogenic effects of XN on 3T3-L1 adipocytes

To identify the possible mechanism underlying the beiging effect of XN, the selective AMPK inhibitor, dorsomorphin, was used to determine UCP1 protein expression levels. When mature 3T3-L1 adipocytes were treated with XN 25 μM, results showed an increase in UCP1 expression. However, this effect was reversed by co-incubation of dorsomorphin with XN, suggesting that XN-induced beiging is mediated via AMPK signaling pathway (Fig. 5). Combined treatment with XN and AICAR resulted in a potentiated effect in UCP1 expression in comparison to XN or AICAR treatment alone.

### XN regulates adipogenesis and lipolysis through the AMPK pathway

**Fig. 6:**
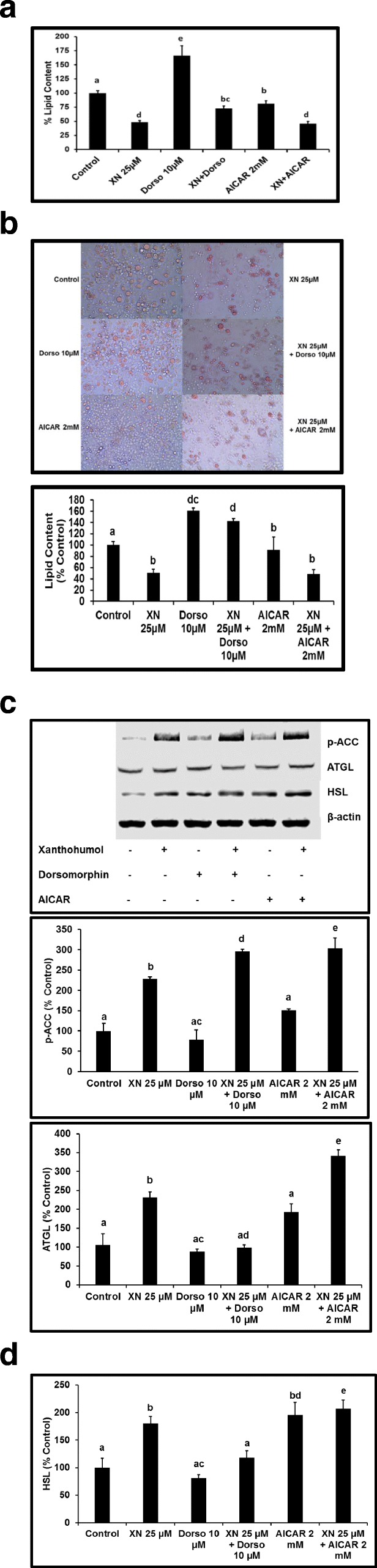
XN-induced inhibition of adipogenesis and stimulation of lipolysis is reversed in the presence of AMPK inhibitor, dorsomorphin. **a** 3T3-L1 preadipocytes were treated on day 0 until day 7 of maturation and lipid accumulation was quantified by AdipoRed™ assay. **b** Differentiated, mature adipocytes were stimulated with XN, dorsomorphin, or AICAR on day 10 for 72 h and Oil Red O staining was performed to measure lipid content. **c** Whole cell lysates were extracted and Western blotting experiments conducted to quantitate the expression of HSL (*6c*), ATGL (*6c*), and p-ACC (*6c*) protein levels post 1 h XN, dorso, or AICAR stimulation. All data are presented as the mean ± SEM. Means denoted with different letters are statistically different, *P* < 0.05

Literature suggests that AMPK is crucial for the mediation of preadipocyte maturation [31]. To investigate the effects of AMPK on XN-induced inhibition of adipogenesis, maturing preadipocytes were treated with test compounds for 0–7 days. AdipoRed™ assay results showed that XN decreased lipid content by 49% ± 6 when compared to the control. AICAR treatment alone decreased lipid content by 8% ± 22 when compared to the control. To the contrary, XN in the presence of AICAR decreased lipid content by 51% ± 7. Dorsomorphin increased lipid content significantly by 61.5% ± 4.29. Noteworthy, XN + dorsomorphin increased lipid content by 42.25% ± 4.78 (Fig. 6a) indicating the reversal of XN-induced inhibition on adipogenesis.

Lipolysis implies the breakdown of triacylglycerols in adipocytes and subsequent release of glycerol and fatty acids. Increased expression of adipose triglyceride lipase (ATGL) and hormone-sensitive lipase (HSL), key enzymes involved in the intracellular degradation of triacylglycerols, suggests augmented lipolysis. In our study, XN increased the expression of HSL and ATGL indicating an increase in lipolysis. Interestingly, in the presence of dorsomorphin, XN decreased the expression of both HSL and ATGL indicating the role of AMPK in XN-induced lipolysis (Fig. 6c). Moreover, AMPK regulates lipid metabolism via the phosphorylation and subsequent inactivation of ACC, the rate limiting enzyme involved in fatty acid biosynthesis. Here, we report that XN stimulates the phosphorylation of ACC (Fig. 6c), notably, in the presence of AICAR, we observed significant potentiated effects, further supporting its role in fatty acid oxidation mediated through the AMPK pathway.

## Discussion

The current study provides novel insights into the anti-obesity effects of XN and the signaling pathways involved. We have successfully demonstrated that mature 3T3-L1 adipocytes stimulated with XN induces the beige phenotype and drives thermogenic programming through significant upregulation of beige-fat specific proteins such as UCP1, PGC-1α, TBX-1, and CIDE-A. TBX-1 is a beige-fat specific marker and beige adipocytes are highly enhanced with TBX-1 [6]. Similarly, the thermoregulatory marker CIDE-A has been shown to be enhanced in BeAT and is considered a beige marker [32]. In contrast, ZIC1 is preferentially expressed in brown adipocytes [33]. Increased expression of UCP1 increases glycolytic rate due to reduced efficiency of ATP formation [34]. Here we report that XN-induced upregulation of UCP1 expression is accompanied by an increase in the oxygen consumption rate.

A key characteristic of brown fat and beige fat is the increased numbers of mitochondria [35]. Our data suggests that XN significantly increases mitochondrial biogenesis in mature 3T3-L1 adipocytes accompanied by the upregulation of PGC-1α, the central regulator of mitochondrial biogenesis and thermogenic programming [36]. Additionally, UCP1 expression was elevated in 3T3-L1 adipocytes upon stimulation with XN, confirming mitochondria oxidation and the acquisition of thermogenic properties [37].

XN was reported to induce apoptosis in mature adipocytes and inhibit adipogenesis in the mouse 3T3-L1 adipocyte cell line [38]. To the contrary, Mendes and colleagues claimed that XN does not improve the metabolic profile linked to obesity [39]. Our results are in agreement with Yang et al. [38], findings and confirm the anti-adipogenic effects of XN under in vitro conditions.

Previous studies provide evidence that pharmacological and genetic approaches to activate AMPK may improve the metabolic profile [40–42]. Therefore, activation of the AMPK signaling pathway is crucial to achieving and maintaining energy homeostasis [43]. Our data demonstrates that XN significantly upregulates the expression of p-AMPK, and this effect is reversed with the inhibition of p-AMPK. Flavonoids such as curcumin, chrysin, and quercetin induce the brown-like phenotype in 3T3-L1 adipocytes [7, 44, 45]. In particular, resveratrol-induced beiging in inguinal WAT was mediated through the activation of AMPKα1 [4]. Likewise, curcumin-induced browning was found to be mediated through the activation of the AMPK pathway [7]. In this study, we have shown that XN significantly increased the expression of the thermogenic protein UCP1, and this upregulation is mediated partly through the activation of the AMPK pathway as evidenced by the arrest of UCP1 expression with XN plus dorsomorphin.

The anti-adipogenic effects of flavonoids have been suggested to be regulated via the AMPK signaling pathway in 3T3-L1 adipocytes [46, 47], however, the role of AMPK in regulating lipolysis has been controversial. Yin et al., reported that AMPK activation is essential in promoting lipolysis in vitro [48] while in vivo data demonstrated that AMPK activation is anti-lipolytic [49]. Our data shows that XN suppressed adipocyte differentiation, reduced the accumulation of lipid content and lipid droplet size, increased oxygen consumption rates, and increased enzymes involved in fatty acid oxidation. Noteworthy, this anti-adipogenic and lipolysis effect of XN was abolished in the presence of dorsomorphin. These results are in agreement with the effects of curcumin in the presence of dorsomorphin [7], confirming the role of AMPK in phytochemical-induced lipolysis in adipocytes. Furthermore, we incubated cells with XN plus AICAR with the goal of demonstrating additive or synergistic effects. Combined treatment of XN with AICAR resulted in potentiated HSL, ATGL, p-AMPK/AMPK, UCP1, and p-ACC expression. However, no potentiated inhibition of adipogenesis was noticed with XN plus AICAR and warrants further investigation. Activation of AMPK by XN represents a novel approach to anti-obesity therapies, and suggests a role for AICAR in combination therapies.

Several pathways have been identified as being required in the beiging of adipocytes, including the mTOR signaling pathway [50]. Interestingly enough, the activation of AMPK inhibits mTOR and crosstalk between these two pathways has been studied. While AMPK activation is crucial to the brown adipocyte differentiation process and functioning in brown and beige fat [50–53], inhibition of mTOR signaling during the earlier stages of differentiation attenuates brown adipogenesis and cell number, suggesting that brown adipogenesis is dependent upon mTOR activation [50]. In contrast, pharmacological inhibition of AMPK with the indirect inhibitor iodotubercidin at days 0 or 5 blocked brown adipocyte differentiation while this inhibition during days 7 to 10 increased lipid accumulation [50]. This data suggests a negative crosstalk between the mTOR/AMPK pathways essential throughout the beiging of WAT.

Taken together, these results provide a novel insight into the molecular mechanism behind XN’s multi-faceted anti-obesity effects. Notably, the XN-induced activation of AMPK results in the beiging of mature 3T3-L1 adipocytes, enhanced lipolysis and inhibition of adipogenesis.

## Conclusion

In conclusion, findings from this study demonstrate that XN possesses anti-obesity effects like inhibition of adipogenesis, induction of beiging and lipolysis, making for an attractive pharmacological drug therapy for the treatment and prevention of obesity. In vitro studies using 3T3-L1 and primary human adipocytes provide novel evidence of XN’s role in the induction of beiging and the signaling pathways involved.

## Additional file

Additional file 1: Full blots for the Westerns are provided in the supplementary files. (PPTM 532 kb)

## Abbreviations

ACC: Acetyl-CoA carboxylase
AICAR: 5-Amionimidazole-4-carboxamide
AMPK: AMP-activated protein kinase
ATGL: Adipose triglyceride lipase
BAT: Brown adipose tissue
BeAT: Beige adipose tissue
CIDE-A: Cell death-inducing DFFA-like effector a
CS: Calf serum
Dexa: Dexamethasone
DM I/II: Differentiation media I/II
DMEM: Dulbecco’s Modified Eagle’s medium
DMSO: Dimethyl sulfoxide
Dorso: Dorsomorphin
FBS: Fetal bovine serum
HSL: Hormone sensitive lipase
IBMX: Isobutylmethylxanthine
Ins: Insulin
Iso: Isoproterenol
mTOR: Mechanistic target of rapamycin
OCR: Oxygen consumption rate
p-ACC: Phosphorylated acetyl-CoA carboxylase
p-AMPK: Phosphorylated AMP-activated protein kinase
PGC-1α: Peroxisome proliferator-activated receptor gamma coactivator 1-alpha
PS: Penicillin streptomycin
PVDF: Polyvinylidene difluoride
Rosi: Rosiglitazone
TBX-1: T-Box transcription factor 1
TNF-α: Tumor necrosis factor alpha
UCP1: Uncoupling protein 1
WAT: White adipose tissue;
XN: Xanthohumol
ZIC1: Zinc finger protein 1
β-actin: Beta actin
