# Supplementary material for: Novel anti-obesity effects of beer hops compound xanthohumol: role of AMPK signaling pathway
Source: Nutr Metab (Lond). 2018 Jun 15;15:42. doi: 10.1186/s12986-018-0277-8 (PMC6003190; doi:10.1186/s12986-018-0277-8)
Supplement: Supplementary file 1 — Full blots for the Westerns are provided in the supplementary files. (PPTM 532 kb) [file 12986_2018_277_MOESM1_ESM.pptm]

## Slide 1
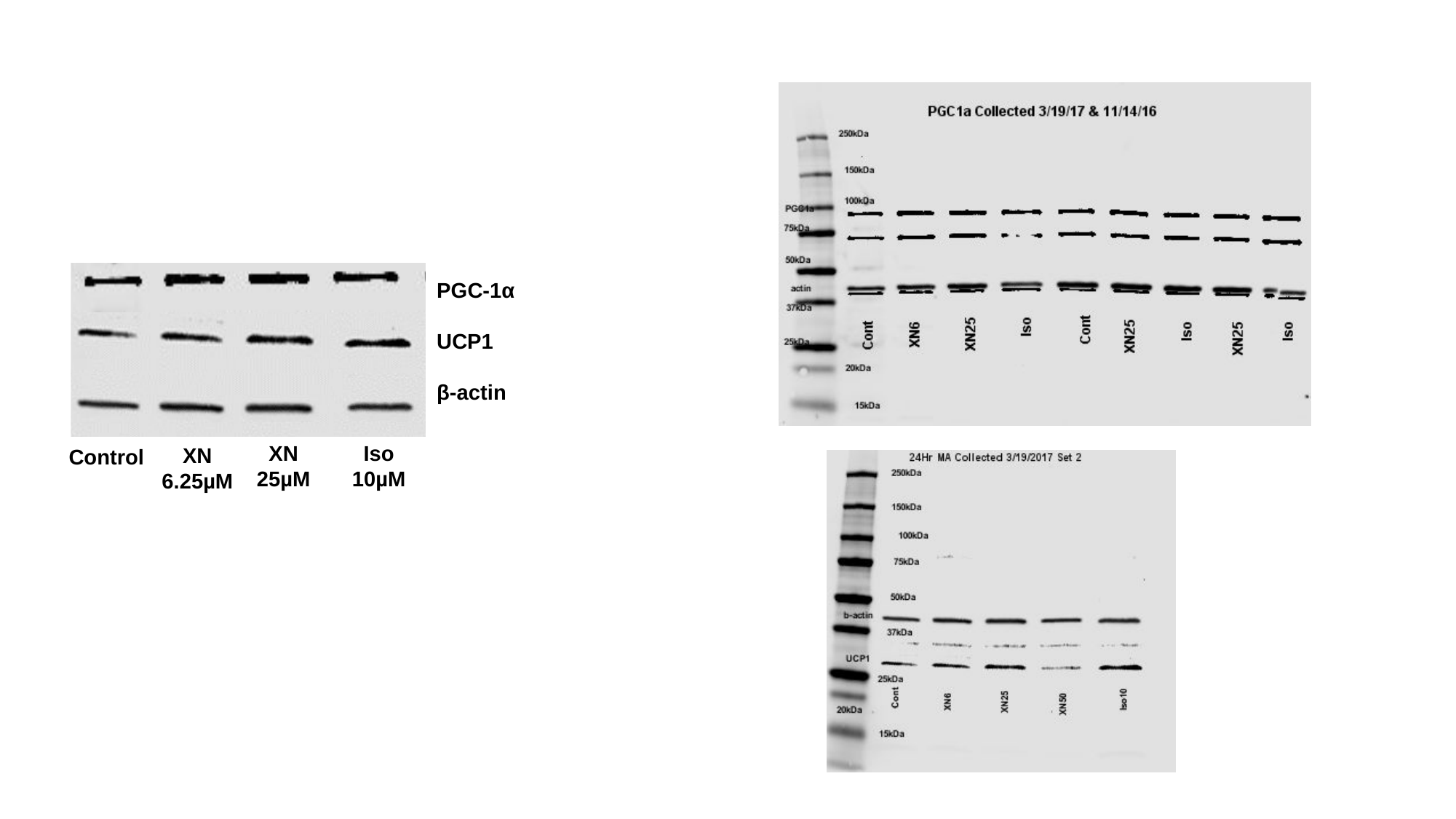

PGC-1α
UCP1
β-actin
Iso
10µM
XN
25µM
XN
6.25µM
Control

## Slide 2
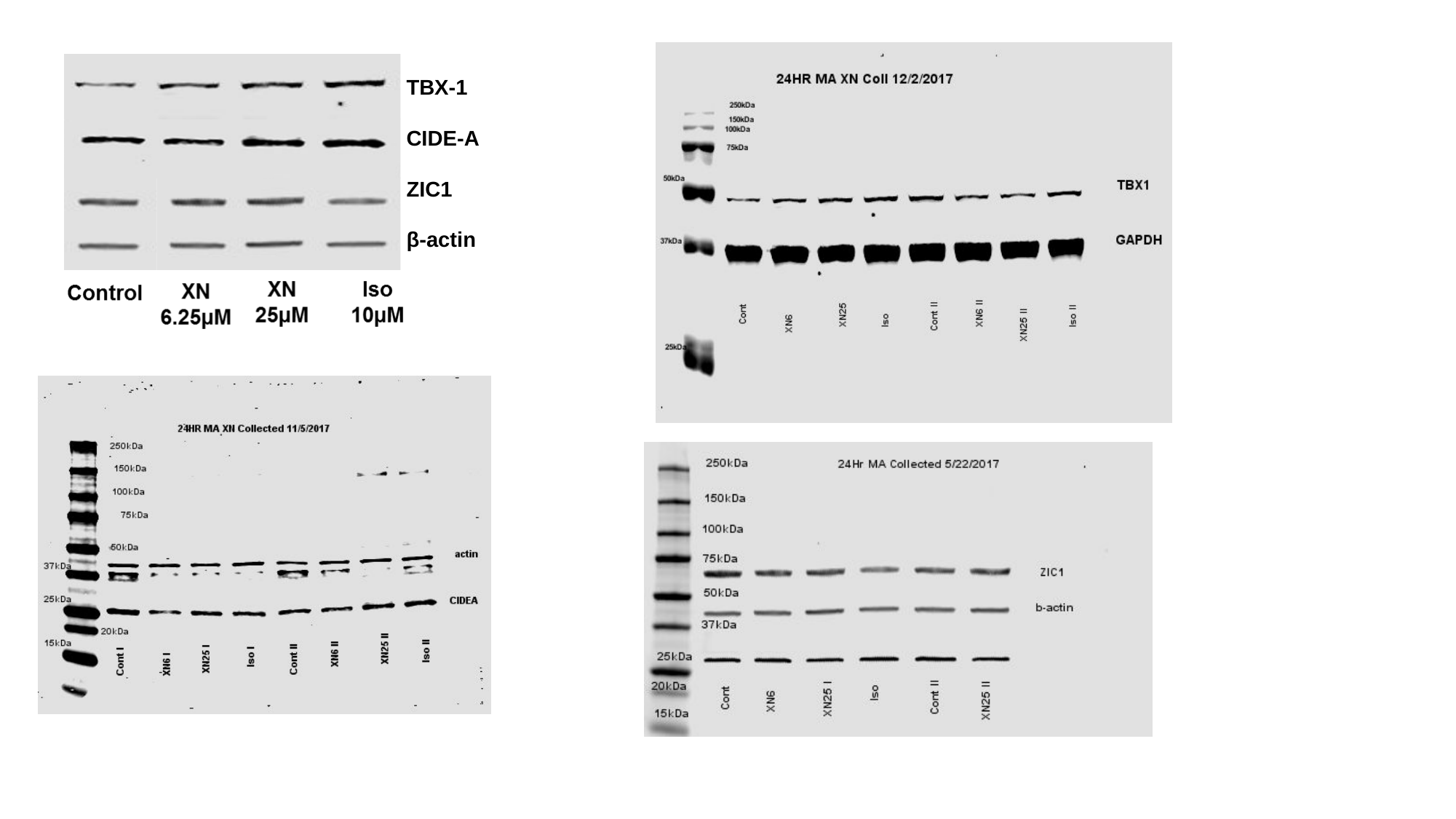

TBX-1
CIDE-A
ZIC1
β-actin

## Slide 3
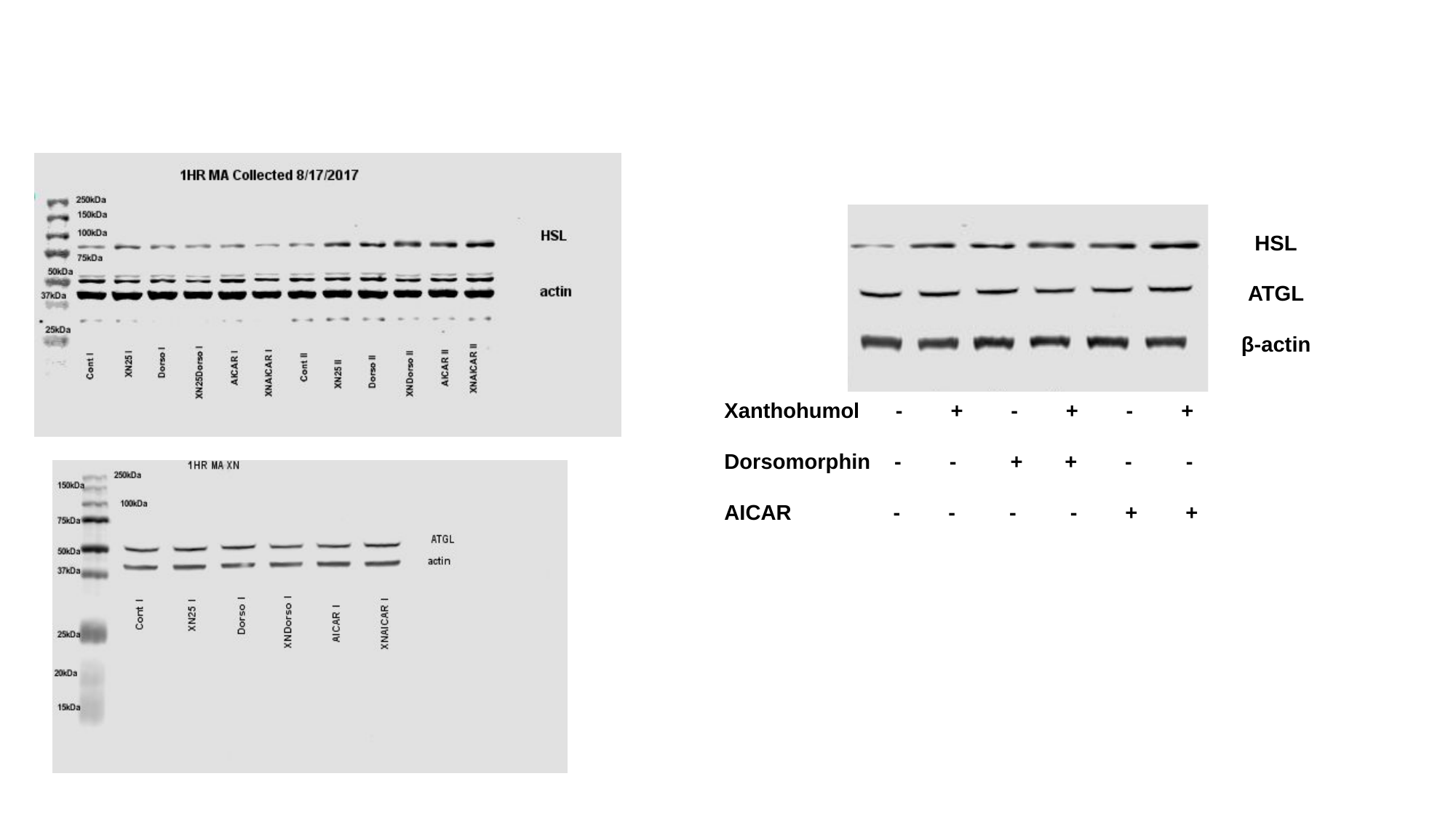

HSL
ATGL
β-actin
Xanthohumol - + - + - +
Dorsomorphin - - + + - -
AICAR - - - - + +

## Slide 4
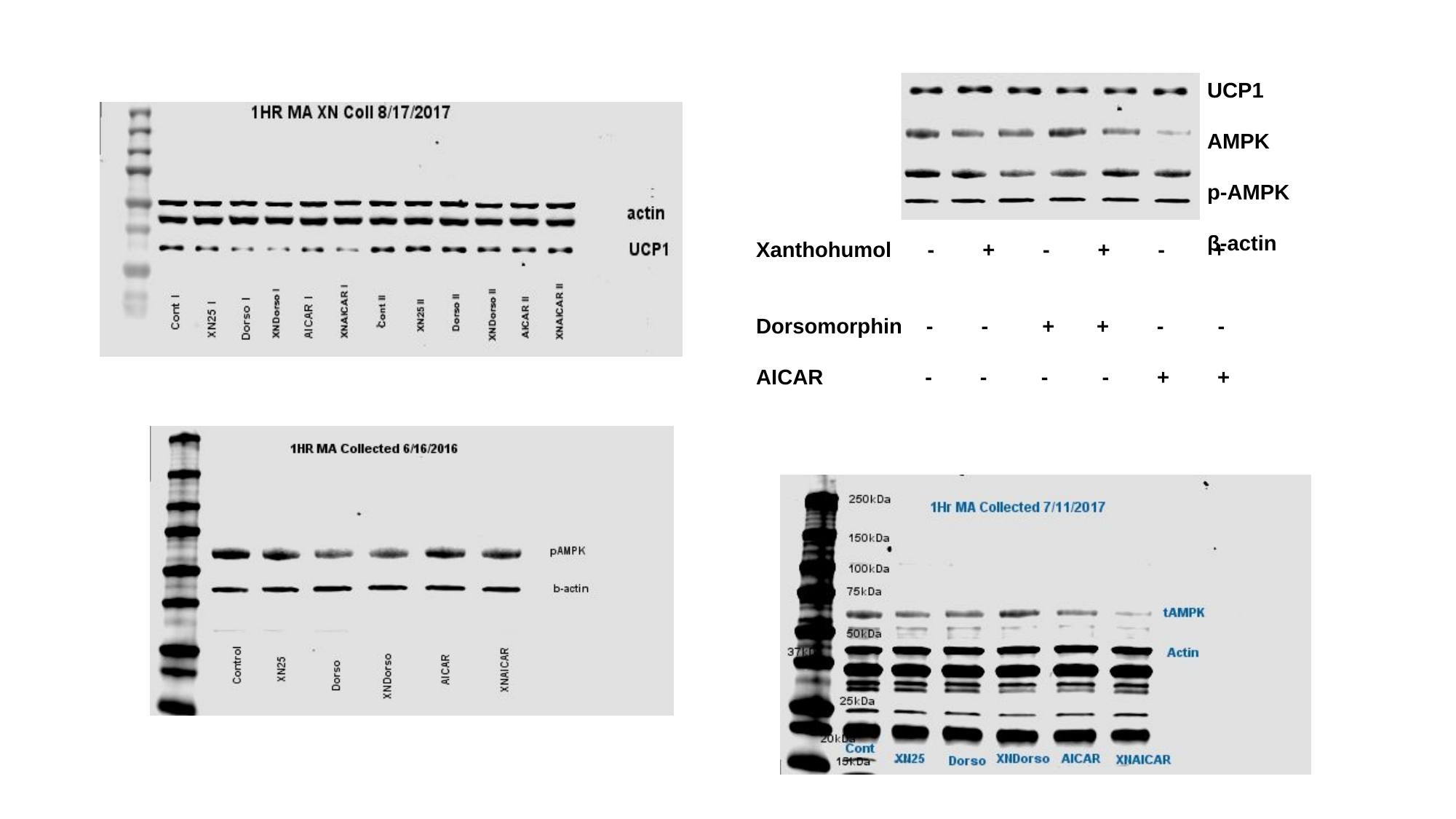

UCP1
AMPK
p-AMPK
β-actin
Xanthohumol - + - + - +
Dorsomorphin - - + + - -
AICAR - - - - + +
